# Drive the Car(go)s—New Modalities to Control Cargo Trafficking in Live Cells

**DOI:** 10.3389/fnmol.2017.00004

**Published:** 2017-01-20

**Authors:** Payel Mondal, John S. Khamo, Vishnu V. Krishnamurthy, Qi Cai, Kai Zhang

**Affiliations:** ^1^Department of Biochemistry, University of Illinois at Urbana-ChampaignUrbana, IL, USA; ^2^Neuroscience Program, University of Illinois at Urbana-ChampaignUrbana, IL, USA; ^3^Center for Biophysics and Quantitative Biology, University of Illinois at Urbana-ChampaignUrbana, IL, USA

**Keywords:** synaptic transmission, neurological disorders, cargo trafficking, motor proteins, axonal transport, optogenetics, chemically induced dimerization, photoactivatable proteins

## Abstract

Synaptic transmission is a fundamental molecular process underlying learning and memory. Successful synaptic transmission involves coupled interaction between electrical signals (action potentials) and chemical signals (neurotransmitters). Defective synaptic transmission has been reported in a variety of neurological disorders such as Autism and Alzheimer’s disease. A large variety of macromolecules and organelles are enriched near functional synapses. Although a portion of macromolecules can be produced locally at the synapse, a large number of synaptic components especially the membrane-bound receptors and peptide neurotransmitters require active transport machinery to reach their sites of action. This spatial relocation is mediated by energy-consuming, motor protein-driven cargo trafficking. Properly regulated cargo trafficking is of fundamental importance to neuronal functions, including synaptic transmission. In this review, we discuss the molecular machinery of cargo trafficking with emphasis on new experimental strategies that enable direct modulation of cargo trafficking in live cells. These strategies promise to provide insights into a quantitative understanding of cargo trafficking, which could lead to new intervention strategies for the treatment of neurological diseases.

## Introduction

The human brain has approximately 86 billion neuronal cells (Azevedo et al., 2009), each of which possesses a large number of synapses to other cells. For instance, each neocortical neuron has an average of about 7000 synapses for exchanges of information (Pakkenberg et al., 2003). Synaptic transmission, which relays information from one cell to the next via coupled events between electrical and chemical signals through synapses, plays a crucial role in learning and memory consolidation. In response to cell depolarization (electrical signals), neurotransmitters (chemical signals) are released into the synaptic cleft, bind to their postsynaptic receptors and trigger downstream signaling activities in the postsynaptic cell. Defective synaptic transmission causes a variety of neurological disorders (van Spronsen and Hoogenraad, 2010) and cognitive diseases (Lau and Zukin, 2007; Südhof, 2008).

Quantitative analysis of synapses has found that, in addition to neurotransmitters, many other molecules and organelles are enriched in the synapse. These molecules include receptors and channels, cytoskeleton, kinases and phosphatases and their regulators, GTPases and their regulators, motor proteins, scaffolding proteins, components of signaling and membrane trafficking, and mitochondria (Ziv and Garner, 2004; Sheng and Hoogenraad, 2007; Bourne and Harris, 2008; Margeta et al., 2008). Indeed, proteomic studies have revealed that more than 2000 different proteins reside in the synapse (Dieterich and Kreutz, 2016). A portion of these synaptic components are likely to be synthesized locally, given that components of translation machinery, e.g., polyribosomes, reside in dendritic shafts and spines. Indeed, it has been shown that rapid dendritic protein synthesis occurs during metabotropic glutamate receptor (mGluR)-dependent long-term depression (LTD; Huber et al., 2000). De-centralized protein synthesis has been an emerging paradigm in studying how neurons achieve specific functions and signals that occur in highly compartmentalized subcellular domains (Holt and Schuman, 2013). Many crucial synaptic components, however, cannot be produced locally in the synapse (Kennedy and Ehlers, 2006). These components include neurotrophin receptors (Ascaño et al., 2009), dense core vesicles, synaptic vesicle precursors (Goldstein et al., 2008) and neurotransmitter receptors (Kneussel and Loebrich, 2007; Shepherd and Huganir, 2007). Biogenesis of these macromolecules often occurs at a great distance from the synapse. The extremely polarized neuronal morphology excludes access of these macromolecules to synapses based on diffusion. As a result, these synaptic components require energy-consuming, motor protein-driven trafficking mechanism to reach their sites of action (Schlager and Hoogenraad, 2009). Thus, a functional synapse requires properly regulated cargo trafficking.

In this review article, we discuss basic cargo trafficking machinery with emphasis on the recently developed strategies that allow active manipulation of cargo trafficking in live cells. Here, cargo trafficking is defined as the process that involves motor-protein-driven transport along cytoskeletons, in contrast to trafficking involved in neuronal activity-regulated cargo endocytosis, exocytosis or lateral diffusion within the plasma membrane. The biotechnological advances discussed herein promise to generate new insights into the understanding of synapse building, synaptic transmission and neurological diseases.

## Basic Components of Cargo Trafficking

Cargo trafficking is mediated through the interaction of the vesicle with the cytoskeletal tracks. Basic components of cargo trafficking include cargos (vehicles), motor proteins (wheels), cytoskeleton (road) and energy (fuel).

### Cargos

In axons, cargos travel through either fast or slow axonal transport (Vallee and Bloom, 1991). Cargos that travel through fast axonal transport have an average speed of about 0.5–5 micron/s (40–400 mm/day). Synaptic vesicles and enzymes for neurotransmitter metabolism use anterograde transport (from the cell body to the axon terminal); internalized membrane receptors and neurotrophins use retrograde transport (from the axon terminal to the cell body); organelles such as mitochondria travel in both anterograde and retrograde directions through engagement of motor adaptor proteins such as trafficking kinesin protein (TRAK)/Milton (van Spronsen et al., 2013). Cargos that travel through slow axonal transport have an average speed of 0.3–8 mm/day. These cargos include “building materials” of neuronal cytoskeletons such as neurofilaments and microtubules, actins, spectrin and tau proteins. Both fast and slow axonal transport adopts a “stop-and-go” pattern, i.e., cruising intersected by pausing (Brown, 2000). Intriguingly, the slow axonal transport is driven by “fast” motors, and the slow speed is due to prolonged pauses (Brown, 2003).

### Cytoskeleton and Motor Proteins

Cargo trafficking depends on the interaction between cytoskeleton and motor proteins (Vale, 2003). The neuronal cytoskeleton is composed of microtubules, actin filaments and neurofilaments (Kevenaar and Hoogenraad, 2015). The main function of neurofilaments, enriched primarily in axons, is to control the axon diameter and axonal conductance (Yuan et al., 2012). Microtubules and actin filaments serve as tracks for motor proteins in axonal and dendritic shafts. On microtubules, kinesin superfamily motor proteins drive anterograde transport (Gennerich and Vale, 2009; Hirokawa et al., 2009); cytoplasmic dyneins drive retrograde transport. One type of motor protein can transport a variety of cargos. For instance, kinesin-1 transports components of cytoskeleton, mitochondria and Soluble NSF Attachment Protein Receptor (SNARE) proteins (Hirokawa and Noda, 2008). Such a specificity of transport can be achieved through splice variants of motor proteins (Cyr et al., 1991) or post translational modifications such as selective phosphorylation in kinesin light chain (Ichimura et al., 2002; Vagnoni et al., 2011). Myosins are a superfamily of motor proteins that travel along actin filaments (Mitchison and Cramer, 1996; Blanchoin et al., 2014). Recently, super-resolution microscopy showed that axonal actin is also organized in regularly spaced rings that wrap around the circumference of axons (Xu et al., 2013). This subpopulation of axonal actin is likely to provide mechanical support for the axon membrane and may not be involved in the myosin-dependent cargo trafficking.

### Energy

Intriguingly, although mitochondria are the major organelles that provide energy to boost up molecular machineries in cells (Sheng, 2014), they may not be the energy resource for axonal transport. Instead, the energy is more likely to be supplied by ATP generated by vesicular glycolysis (Zala et al., 2013). Inhibition of ATP production from mitochondria via oligomycin, an inhibitor of mitochondrial H^+^-ATP-synthase, did not affect the fast axonal transport of brain-derived neurotrophic factor (BDNF). In contrast, treating cells with iodoacetate, which inhibits glyceraldehyde-3-phosphate dehydrogenase (GAPDH), the key glycolytic enzyme, significantly reduced the average velocity of BDNF (Zala et al., 2013). On the other hand, although oligomycin had no effect on vesicle transport, it blocked mitochondria trafficking, consistent with previous findings that loss of mitochondrial ATP production induces loss of mitochondrial dynamics (Kaasik et al., 2007).

## Tracking Cargo Transport in Live Cells

Early work used radioactive labeling to detect cargo trafficking in neurons (Lasek, 1967; Ochs et al., 1969). Although this method confirmed the existence of fast and slow axonal transport, it lacked the resolution to track individual cargos. Video-enhanced contract-differential interference contrast microscopy (Brady et al., 1982) allowed for tracking of individual cargoes, but could not differentiate their identities. Advanced fluorescence microscopy techniques, such as single-molecule fluorescence microscopy, have enabled real-time tracking of neurotrophin transport in live neuronal cells (Tani et al., 2005). However, the data acquisition time was limited to tens of seconds owing to photobleaching of the organic fluorophore such as Cy3. More photostable probes such as semiconductor nanocrystals (quantum dots) allowed for continuous tracking of cargos along neuronal processes for several minutes over hundreds of microns (Cui et al., 2007). Although typical quantum dots (about 20 nm in diameter) are much larger than organic fluorophores, they did not seem to disturb the biological activity of neurotrophin (Cui et al., 2007). Recent development of small quantum dots (9 nm in diameter) will further improve their use in live cell imaging (Cai et al., 2014). A critical difference between *in vitro* and live-cell cargo trafficking is that intracellular trafficking is regulated not only by motor proteins, but also by cargo-organelle and cargo-cytoskeletal interactions. Evidence shows that early endosomal interaction with microtubule intersection, other early endosomes, and endoplasmic reticulum contributes to the pausing of epidermal growth factor-containing early endosomes (Zajac et al., 2013). To effectively determine the directionality of cargo trafficking, Campenot (1977) designed the first prototype of compartmentalized culturing device that separates the cell body from the distant neurite. Improved quality has been achieved by replacing Teflon with polydimethylsiloxane (PDMS), a transparent and highly biocompatible material (Taylor et al., 2005; Mudrakola et al., 2009; Zhang et al., 2011).

## Restoration of Cargo Trafficking Exerts Neuroprotective Effects

Defective cargo trafficking has been found in a variety of neurological disorders (Tischfield et al., 2011) and brain injury (Povlishock and Jenkins, 1995). For instance, huntingtin-associated protein 1 (HAP1) is highly expressed in neurons and mediates kinesin-based anterograde transport (McGuire et al., 2006). In Huntingtin disease, stronger interaction between huntingtin protein and HAP1 leads to detachment of molecular motors from BDNF-containing cargos and reduced BDNF transport (Charrin et al., 2005). Analysis of axonal transport defects in human disease has been comprehensively reviewed and will not be repeated here (Roy et al., 2005; Chevalier-Larsen and Holzbaur, 2006; De Vos et al., 2008; Morfini et al., 2009; Hirokawa et al., 2010; Hinckelmann et al., 2013). Notably, although the causality of defective cargo trafficking to neurological disorders is still under debate (Goldstein, 2012), multiple lines of research have provided evidence that restoration of axonal transport can exert neuroprotective effects (Hinckelmann et al., 2013). For instance, failed retrograde transport of nerve growth factor (NGF) from the hippocampus to the basal forebrain caused reduction in size and number of basal forebrain cholinergic neurons (BFCN) in the partial trisomy 16 (Ts65Dn) mouse model of Down’s syndrome. Such defects were rescued by delivering NGF directly to the cell bodies of BFCN through intracerebroventricular administration, which bypassed defective axonal transport (Cooper et al., 2001). Reduction of the endogenous level of Tau, a microtubule-associated protein, ameliorated amyloid β-induced deficits in an Alzheimer’s disease mouse model (Roberson et al., 2007). Tau reduction has also been shown to rescue defective axonal transport of mitochondria and neurotrophin receptors (Vossel et al., 2010). Modulation of tau-microtubule interactions has been proposed as a therapeutic strategy for the treatment of tauopathies (Ballatore et al., 2011). The majority of these studies used an indirect way (e.g., bypassing axonal transport or genetic modulation of microtubule-association protein) to rescue defective transport. It remains unknown if direct rescuing of cargo trafficking is sufficient to induce neuroprotective effects. Recent biotechnological advances have started to offer new opportunities to address this issue.

## Direct Control of Cargo Trafficking in Live Cells

**Figure 1:**
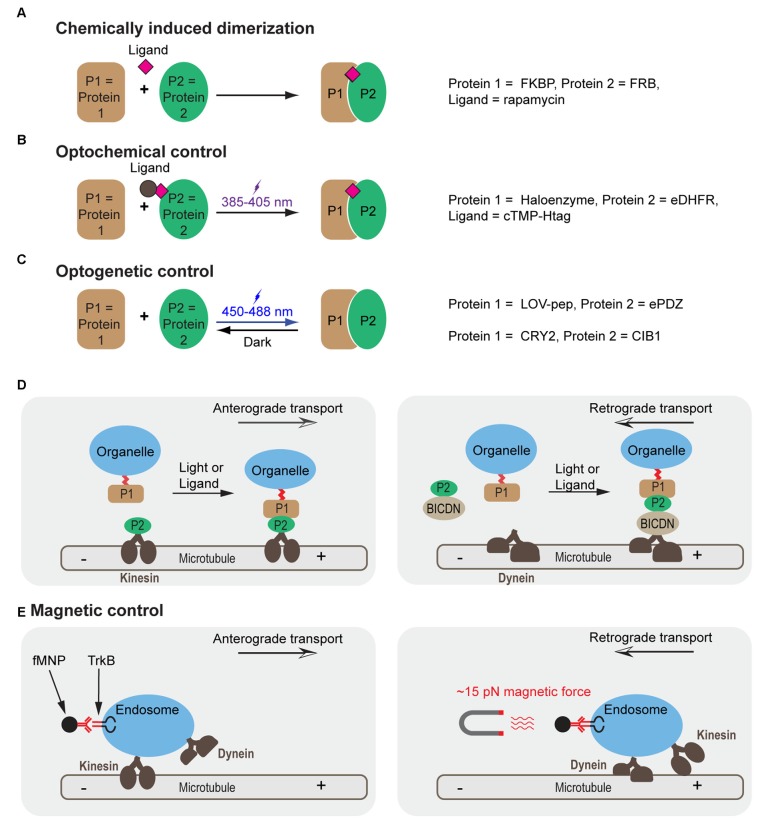
**Molecular mechanisms for controlling cargo tracking in cells. (A–C)** Protein dimerization induced by chemically induced dimerization (CID) **(A)**, optochemical **(B)** and optogenetic **(C)** approaches. **(D)** Cargo trafficking can be controlled by construction of fusion protein between motor proteins (such as kinesin), motor protein adapters (e.g., BICDN) and organelle-targeting signals with different combinations of protein pairs. **(E)** Control of endosome trafficking using magnetic nanoparticles functioned with antibody for TrkB. A force of 15 pN reverses the direction of transport from anterograde to retrograde. FKBP, FK506 Binding Protein; FRB, FKBP Rapamycin Binding domain of mammalian target of rapamycin (mTOR); eDHFR, *Escherichia coli* dihydrofolate reductase, cTMP-Htag, photocaged trimethoprim-Halo tag, photocaged trimethoprim; LOV-pep, light, oxygen, voltage-peptide epitope; ePDZ, engineered PDZ domain; CRY2, *Arabidopsis* cryptochrome 2; CIB1, cryptochrome 2 interacting basic helix-loop-helix; BICDN, the amino terminus of bicaudal D homolog 2 (BICD2); TrkB, tropomyosin-related kinase B; fMNP, anti-TrkB functionalized superparamagnetic nanoparticle.

Correct positioning of organelles plays a crucial role in signaling regulation, cell differentiation and development (van Bergeijk et al., 2016). For instance, localized positioning of endosomes contributes to polarization and local outgrowth of neuronal cells (Sadowski et al., 2009; Eva et al., 2010, 2012; Golachowska et al., 2010; Higuchi et al., 2014). Similarly, correct mitochondrial positioning helps in axon branching (Courchet et al., 2013; Spillane et al., 2013) and synaptic function (MacAskill et al., 2010; Sheng and Cai, 2012). Golgi positioning is crucial to axon specification and dendrite development (Yadav and Linstedt, 2011; Ori-McKenney et al., 2012). Active nuclear positioning ensures correct cellular function during cell division, migration and differentiation (Gundersen and Worman, 2013). Altered positioning of dynamic organelles in cells is involved in neurodegenerative disorders. For instance, perinuclear accumulation of lysosomes is increased in a cellular model of Huntington’s disease (Erie et al., 2015). Taking advantages of accumulating knowledge of motor and scaffolding proteins involved in organelle transport (Fu and Holzbaur, 2014), emerging new biotechnologies have enabled direct control of organelle trafficking in live cells with high spatiotemporal resolution and cargo specificity (Figure 1 and Table 1).

**Table 1:** **Summary of current controlling mechanisms for cargo trafficking in live cells**.

Controlling mechanism	Controlling module	Controlled cargo	Model system	References
**Trafficking along cytoskeletons**
Chemical	FKBP-FRB	Peroxisome	COS-7 and MRC5 cells	Kapitein et al. (2010)
	FKBP-FRB	Endosome	Rat embryonic fibroblast cells	Bentley et al. (2015)
Optochemical	Haloenzyme-eDHFR	Mitochondria and peroxisome	HeLa cells	Ballister et al. (2015)
	Haloenzyme-eDHFR	Peroxisome	HeLa cells	Olenick et al. (2016)
Optogenetic	LOV-PDZ	RAB11 positive endosome	COS-7 cells	van Bergeijk et al. (2015)
	CRY2PHR-CIBN	Mitochondria and peroxisome	COS-7 cells	Duan et al. (2015)
	LOV2	Myosin and kinesin	*In vitro*	Nakamura et al. (2014)
Magnetic	Electromagnetic needle-fMNP	TrkB-containing endosome	Retinal ganglion cells	Steketee et al. (2011)
**Trafficking between intracellular compartments**
Chemical	FM-ligand	Insulin and growth hormone	HT1080 cells and mice	Rivera et al. (2000)
	FM-Shield-1	Transferrin receptor, VSVG, NgCAM, GluR1, mGluR2	Cortical neurons	Al-Bassam et al. (2012)
	Biotin-streptavidin	Proteins with targeting signal	HeLa cells	Abraham et al. (2016)
Optogenetic	UVR8	VSVG	HEK293T, COS-7, hippocampal neurons	Chen et al. (2013)

### Chemically Induced Dimerization (CID)

Chemically induced dimerization (CID) uses a small molecule to induce binding between two proteins (Putyrski and Schultz, 2012; Rakhit et al., 2014; Voss et al., 2015; Figure 1A). A commonly used module is the rapamycin based FK506 Binding Protein (FKBP) and the FKBP Rapamycin Binding (FRB) domain of mammalian target of rapamycin (mTOR; Banaszynski et al., 2005; Inoue et al., 2005). This system has been used to recruit motor proteins (or their adapters) to peroxisomes to achieve rapamycin-induced transport along corresponding cytoskeletons (Kapitein et al., 2010). A similar scheme has also been used to position early endosomes or late endosomes by fusing the FKBP-FRB system to endosomal markers (Rab5 and Rab7) and motor proteins (Bentley et al., 2015).

### Optochemical Control

An optochemical system utilizes a photoactivatable ligand to induce association of a pair of proteins (Figure 1B). One such ligand is cTMP-Htag, a synthetic, cell-permeant, small molecule comprising a Halotag ligand (a ligand for Haloenzyme) linked to photocaged trimethoprim (TMP), a ligand for *Escherichia coli* dihydrofolate reductase (eDHFR). A pulse of UV light uncages TMP and fully activates the dual-ligand, which crosslinks the Haloenzyme and the eDHFR-fusion protein (Ballister et al., 2014). When applied in cells where eDHPR was fused to motors or motor effectors and Halotag was fused to cargos, eTMP-Htag enabled light-controlled crosslinking between cargos and motors (Ballister et al., 2015). This system has allowed for directional control of mitochondria or peroxisome trafficking in neurons. Other optochemical systems, such as those based on photocaged rapamycin (Karginov et al., 2011; Umeda et al., 2011), chemically modified abscisic acid (Wright et al., 2015; Zeng et al., 2015) and gibberellic acid (Schelkle et al., 2015), photoactivatable crosslinker for SNAPTag and HaloTag (Zimmermann et al., 2014), are also expected to achieve similar optochemical control.

### Optogenetic Control

Optogenetics harnesses the power of light to modulate protein-protein interactions in live cells (Figure 1C). Shortly after its initial success in controlling neuronal firing (Banghart et al., 2004; Boyden et al., 2005; Deisseroth, 2011), optogenetics has been extended to control other cellular processes such as gene transcription, translation, protein splicing, protein degradation, cell differentiation and cell death. The possibility of modulating signaling pathways and cell functions with high spatiotemporal precision offers an entirely new modality to dissect molecular mechanisms governing cell fate determination (Toettcher et al., 2011; Zoltowski and Gardner, 2011; Tucker, 2012; Kim and Lin, 2013; Tischer and Weiner, 2014; Zhang and Cui, 2015). Photoactivatable proteins have been used in multiple model systems including yeast (Shimizu-Sato et al., 2002; Tyszkiewicz and Muir, 2008; Hughes et al., 2012; Strickland et al., 2012), mammalian cells (Levskaya et al., 2009; Wu et al., 2009; Yazawa et al., 2009; Kennedy et al., 2010; Toettcher et al., 2011; Idevall-Hagren et al., 2012; Mills et al., 2012; Zhou et al., 2012; Bugaj et al., 2013; Grusch et al., 2014; Kim et al., 2014; Lee et al., 2014; Taslimi et al., 2014; Zhang et al., 2014; Hughes et al., 2015; Kawano et al., 2015; Yumerefendi et al., 2016), primary neurons (Chen et al., 2013; Kakumoto and Nakata, 2013; Konermann et al., 2013), *Drosophila* (Boulina et al., 2013), zebrafish embryos (Liu et al., 2012; Motta-Mena et al., 2014; Buckley et al., 2016) and *Xenopus* embryos (Krishnamurthy et al., 2016). To control cargo trafficking, photoactivatable proteins such as the light, oxygen, voltage-peptide epitope (LOV-pep) and engineered PDZ domain (ePDZ; van Bergeijk et al., 2015) or cryptochrome 2 (CRY2) and cryptochrome 2 interacting basic helix-loop-helix (CIB1; Duan et al., 2015) were fused to cargoes and motor proteins or motor adapters (Figure 1D). Interestingly, directionality of transport seems to depend on the load of motor proteins. By engineering the LOV domain into the lever arm of myosin or kinesin, the directionality of these motor proteins can be reversibly modulated as reported in a recent *in vitro* assay (Nakamura et al., 2014).

### Magnetic Control

Another strategy utilizes magnetic force to reverse cargo transport. Using an electromagnetic needle and antibody-functionalized superparamagnetic nanoparticles (fMNPs), Steketee et al. (2011) could reverse the direction of transport of TrkB-containing endosomes in retinal ganglion cells (Figure 1E). Manipulation of fMNP signaling endosomes by a focal magnetic field altered growth cone motility and halted neurite outgrowth (Steketee et al., 2011).

Notably, trafficking along the secretory pathway between membrane-bound cellular compartments including the endoplasmic reticulum, Golgi apparatus, endosome and plasma membrane can also be controlled via chemical, optochemical and optogenetic strategies. The general strategy involves a chemical- or light-induced activation of the targeting signal, either by uncaging a blocking motif (Abraham et al., 2016) or inducing dissociation of a mislocalized protein cluster (Rivera et al., 2000; Al-Bassam et al., 2012; Chen et al., 2013). Interested readers are encouraged to refer to the references listed in Table 1.

## Outstanding Questions and Future Directions

Cargo trafficking plays a crucial role in neuronal survival, differentiation, axon pathfinding, as well as synaptogenesis and synaptic transmission. With advances in genetic and protein engineering, single-molecule fluorescence microscopy, microfluidics, CID and optogenetics, one can control cargo trafficking with superior spatiotemporal resolution and molecular specificity. Because most of controlling systems are genetically encoded, it is possible to generate novel model systems harboring light- or chemical- sensitive signaling circuits. These tools could thus provide new perspectives to address controversies in the field of cargo trafficking in neuroscience. On the other hand, significant improvement of current technologies is needed before they can be successfully applied in tissues or multicellular organisms. For instance, single-molecule fluorescence microscopy has been mostly applied *in vitro* or in separated cells. Its potential in multicellular organisms has yet to be fully realized, owing to the limited penetration depth of visible light in the high-absorbing, high-scattering biological tissues. Poor penetration of visible light in biological tissues also results in invasiveness and low throughput of current optogenetic techniques, which often relies on insertion of fiber optics or microscale light emitting diodes arrays (Kim et al., 2013) in tissues for light delivery. Successful removal of these technical barriers requires a collaborative effort of researchers from multi-disciplinary fields including physics, material sciences, biochemistry and bioengineering. Shortly after the initial phase of tool development, as demonstrated in recent literature, we believe follow-up work will start to address the signaling outcomes in response to the modulated cargo trafficking. For instance, is defective cargo transport a cause or a result of misregulated neuronal functions and neurological disorders? Can we rescue defective neuronal phenotypes by direct modulation of cargo trafficking? We believe that biotechnological advances will continue pushing forward our understanding of the molecular machinery underlying neuronal survival, differentiation, repair and synaptic transmission and plasticity.

## Author Contributions

PM, JSK, VVK, QC and KZ performed literature search and wrote the initial draft. PM generated Table 1. QC designed and generated Figure 1. QC and KZ wrote the final manuscript.

## Conflict of Interest Statement

The authors declare that the research was conducted in the absence of any commercial or financial relationships that could be construed as a potential conflict of interest.
